# Alloying Elements Effect on the Recrystallization Process in Magnesium-Rich Aluminum Alloy

**DOI:** 10.3390/ma15207062

**Published:** 2022-10-11

**Authors:** Vladimir Aryshenskii, Fedor Grechnikov, Evgenii Aryshenskii, Yaroslav Erisov, Sergey Konovalov, Maksim Tepterev, Alexander Kuzin

**Affiliations:** 1 Institute of Aerospace Engineering, Samara National Research University, Moskovskoye Shosse 34, 443086 Samara, Russia; 2 Departament of Mechanics and Engineering, Siberian State Industrial University, Kirova 42, 654007 Novokuznetsk, Russia

**Keywords:** aluminum, recrystallization, microstructure-property characterization simulation and modeling, heat treatment and surface treatment

## Abstract

This paper addresses the study of the complex effect of alloying elements (magnesium, manganese, copper and zirconium) on changes in magnesium-rich aluminum alloy composition, fine and coarse particle size and number, recrystallization characteristics and mechanical properties. The data obtained made it possible to analyze change in the chemical composition, sizes of intermetallic compounds and dispersoids depending on alloying elements content. The effect of the chemical composition on the driving force and the number of recrystallization nuclei was studied. It was established that the addition of alloying elements leads to grain refinement, including through the activation of a particle-stimulated nucleation mechanism. As a result, with Mg increase from 4 to 5%, addition of 0.5% Mn and 0.5% Cu, the grain size decreased from 72 to 15 µm. Grain refinement occurred due to an increase in the number of particle-stimulated nuclei, the number of which at minimal alloying rose from 3.47 × 10^11^ to 81.2 × 10^11^ with the maximum concentration of Mg, Mn, Cu additives. The retarding force of recrystallization, which in the original alloy was 1.57 × 10^−3^ N/m^2^, increased to 5.49 × 10^−3^ N/m^2^ at maximum alloying. The influence of copper was especially noticeable, the introduction of 0.5% increasing the retarding force of recrystallization by 2.39 × 10^−3^ N/m^2^. This is due to the fact that copper has the most significant effect on the size and number of intermetallic particles. It was established that strength increase without ductility change occurs when magnesium, manganese and copper content increases.

## 1. Introduction

Aluminum is one of the most commonly used metals in modern industry [1,2,3,4,5,6,7,8,9,10,11,12]. Aluminum alloys with a high magnesium content are very popular multifunctional alloys, and are used in the automotive industry, shipbuilding, aerospace engineering and the packaging industry [13,14,15,16,17,18]. The advantages of these alloys include strength, corrosion resistance, weldability and ductility, and the absence of yield plateau [19,20]. Attainment of these characteristics is possible only with the correct choice of parameters of thermomechanical treatment [21,22,23]. Recrystallization is the most important process that must be controlled by the correct choice of modes during thermomechanical treatment of these alloys, since in many respects the features of recrystallization behavior (or its absence) determine the properties of the aluminum alloys listed above [22,24,25]. In addition to parameters of the technological process, the mechanisms of recrystallization are strongly affected by the size and number of intermetallic particles. Finely dispersed particles (less than 1 µm in diameter) inhibit the recrystallization process, sometimes making it impossible due to their large amount [26,27]. Large particles play the role of nuclei in the recrystallized structure according to the mechanism described in the literature as Particle Stimulated Nucleation PSN [28,29].

A number of works have been devoted to the study of size and number of intermetallic particles and their effect on recrystallization in alloys containing magnesium. For example, in [30], alloys AA5182, AA5052 and AA5005 were compared. These alloys were alloyed in various combinations of Mg; Mn; Si; Fe; Cr; Cu and Ti. The number of PSN nuclei for each of the alloys was given, and it was found that the larger the PSN, the smaller the cubic texture, but the research did not show how specific alloying elements affected the number and size of intermetallic particles. In [31], recrystallization was studied on the basis of the 5083 alloy. This alloy contained Mn, Zr, Sc, Cr, Fe, Si and Mg, and the content of the first element remained constant. The effect of other elements on large intermetallic and finely dispersed particles was studied. The influence of the chemical composition on the amount and size of intermetallic compounds, as well as the decelerating force of recrystallization, was not evaluated. In [32], using AA5251 alloy as an example, the relationship between A1(Fe,Mn)Si, Mg_x_Si intermetallics and the recrystallization process, as well as the reduced volume fraction occupied by particles of both phases, were shown. The relationship between intermetallic compounds and the number of nuclei and the retarding force of recrystallization was not described. In [33] a comparison of particles in alloys 5754 and 5182 containing Si, Fe, Cu, Mn, Mg, Cr, Zn and Ti was made, and the number of both large intermetallic and fine particles was described, dependent on microchemistry. However, the research considered the homogenized state and the relationship of these particles, but the recrystallization process was not studied. In [34], the amount number of intermetallic particles was also studied, but their effect on nucleation was not investigated. In [35], the effect of homogenization on the number of fine particles and on the inhibition of recrystallization was studied. Thus, despite a large number of studies devoted to this issue, they have been mainly devoted either to a homogenized state and the connection between particles paying little attention to recrystallization, or to the comparison of two different alloys with several different elements that change at the same time and may not completely coincide. This makes it difficult to analyze their effect on the number and size of intermetallic particles and on nucleation during recrystallization. Therefore, a study of how a gradual increase in the most popular elements in alloying of high-magnesium aluminum alloys affects the size and number of particles, and, consequently, the patterns and mechanisms of the recrystallization process, is an urgent and not fully explored issue. The purpose of this work was to study the influence of magnesium in the range of 4–5%, manganese in the range of 0.2–0.5%, and copper in the range of 0.1–0.5% on intermetallic particles and their influence on nucleation and inhibition of the recrystallization process during annealing of a hot-rolled aluminum alloy. These element concentrations were chosen because they are typical for high magnesium alloys.

## 2. Materials and Methods

An aluminum-magnesium alloy with 4% magnesium content was chosen as the base alloy. During the research, a gradual increase in magnesium (up to 5%), manganese (from 0.2 to 0.5%), copper (from 0.25 to 0.5%) and zirconium (up to 0.05%) was performed. The studied alloys’ chemical composition is presented in Table 1. Ingots sized 35 × 200 × 300 mm^3^ were cast in a metal mold. Experimental melting was performed in a graphite crucible in a medium frequency induction furnace; the molten metal weight was 4–5 kg and the cast ingot weight was 3 kg. The casting temperature was 720–740 °C, the crystallization rate was 2 °C per second, and the rate of cooling after casting was 1 °C per second. Prior to molten metal casting in the casting mold, the alloy was refined by carnallite flux, dosed in proportion of 5 g of flux per 1 kg of charge. After that the segregation layer was skimmed from metal surface. The solidified ingot was removed from the mold and water cooled. The following materials were used to produce the studied alloys: A85 grade prime aluminum, MG90 grade prime magnesium, M1 grade copper, Mn90Al10 grade alloying pellets.

After casting, the ingots were milled on four sides to 30 × 180 × 250 mm^3^ size (to prepare for rolling). After machining the ingots, 8-hour homogenization annealing was performed at 460–480 °C.

Thermo-mechanical modes were calculated for ingot hot rolling. The optimal ingot pre-heat temperature for rolling (450–500 °C) and reduction per pass, depending on the previous pass thickness, were also determined. The hot rolling schedule, enabling us to obtain the required structure, is presented in Table 2. It should be noted that impurities are were the level typical for aluminum alloys with a high magnesium content used in industry. The procedure for the thermomechanical treatment made in the study was: 1. Casting; 2. Homogenization and heating in laboratory furnace; 3. Hot rolling on the laboratory mill; 4. Annealing.

Rolling was performed using a 300 (Dima Maschinen, Esslingen am Neckar, Germany) laboratory mill. A 30 mm thick workpiece was hot rolled to attain a 3 mm thickness (90% total deformation). The maximum force during hot rolling did not exceed 650 kN. NOASAR 8109 rolling oil was used to reduce force and friction during hot rolling.

After hot rolling, the samples were edge-trimmed. The samples were cut to a maximum 300 mm length. They were also marked and annealed at 360 °C for 3 h. The samples were annealed and cooled in a THERMCONCEPT KM 70/06/A laboratory furnace (Thermconcept, Bremen, Germany). After annealing, all samples were cold rolled from 3 mm to 0.3 mm thickness (with equal reductions per pass), which corresponds to 90% total deformation. Cold rolling was performed to obtain the required mechanical properties and target thickness. Rolling forces did not exceed 550 kN during cold rolling.

Mechanical properties of the cold-rolled samples were studied in the longitudinal direction after coating, curing, and pasteurization simulation. The samples were tested using a Zwick/Roell Z050 stretching machine (ZwickRoell, Denmark, Germany) in accordance with EN 541-2006 and EN 10002-1. Five samples per each chemical composition were taken in the longitudinal direction. The samples, ruptured outside the working area during the test, were not accounted for in the final result. The averaged test results are presented below. Mechanical tests results of ultimate yield strength were applied as the criterion for establishing the specific chemical element content during other component additions (Table 1).

Samples for microstructure, electrical conductivity and micro-hardness studies were taken at the end of each processing stage. Micro-hardness and electrical conductivity measurements were carried out because they are very sensitive to changes of the dispersoid fraction, as well as the concentration of the alloying elements in a supersaturated solid solution. It should be noted that electrical conductivity is often used in studies related to aluminum alloys microalloying [36,37,38].

The cross-sectional microstructure of samples was studied in polarized light after section electropolishing in a fluoroboric electrolyte (boric acid—11 g; hydrofluoric acid—30 mL; distilled water—2200 mL). The structure was studied after etching using an Axiovert 40 MAT microscope (Carl Zeiss AG, Oberkochen, Germany). Electrical conductivity was measured using a portable VE-17NTs (LLC NPP “Sigma”, Nalchik, Russia). The excitation current frequency was 100 kHz.

Based on optical microscopy data for samples annealed after hot rolling, the average grain size was determined by the secant method. This was determined only for fully recrystallized samples when magnified to capture 80–200 grains. To determine the average grain size, at least eight secants were chosen in eight areas. An eyepiece-micrometer ruler was used as a secant. Positioning the ruler across the direction of deformation and at an angle of 45°, the number of grains (*n*) intersected by this secant was counted. The average grain size was determined by:

(1) $$D_{cp}=\frac{L\cdot k}{\sum n},$$

where: *L* is the length of the eyepiece-micrometer ruler, mm; *k* is the number of secants, and *n* is the sum of grains intersected by all secants.

In addition to optical microscopy, some samples were studied using automatic analysis of electron backscattered diffraction (EBSD) patterns using a TESCAN VEGA LMH scanning electron microscope with a LaB6 cathode (SEM) equipped with an Oxford Instruments Advanced AZtecEnergy X-ray energy-dispersive microanalysis system and an Oxford Instruments NordLysMax2 EBSD attachment, using AZtec version 2.2 software. For analysis of grain (sub-grain) boundary misorientation, maps were built from areas 690 × 400 µm in size with a scanning step of 0.5 µm. For data collection, a 2 × 2 binning mode was used, providing a high level of structural detail.

Data for analysis were obtained with an indexation coefficient of 95% or more. The error in determining the orientation of the crystal lattice (the average angular deviation between the detected and simulated Kikuchi bands) was no more than ±0.5°. Boundaries with misorientations less 15° were taken as low-angle boundaries. Boundaries with misorientations of more than 15° were considered as high-angle boundaries. When high-angle boundaries were determined, individual grains were defined. For each of them, the misorientation between neighboring point was calculated. If the average misorientation between points was less than 2°, the grain was considered recrystallized and marked as blue. If the average misorientation between points was less than 2°, but subgrain misorentation was also less than 2°, this grain colored yellow and considered as substructed. If the average misorientation between points was more than 2°, the grain was considered deformed and marked in red.

Studies of alloys with additions of 0.5% manganese and 0.5% copper were carried out using a Tecnai G2 F20 S-TWIN TMP transmission electron microscope with a thermal field cathode at an accelerating voltage of 200 kV. A study of the chemical composition of structural components was carried out by energy dispersive spectroscopy (EDS) using an X-Max 80T detector in the energy range 0–10 keV. The energy resolution of the detector was 122 eV.

Micro-hardness was measured to indirectly assess the alloying effect on strength properties. Micro-hardness tests were carried out with a Wolpert 402MVD hardness tester (Wolpert, Bretzfeld, Germany) using the Micro-Vickers method in accordance with GOST 9450-76. The measurements were taken only for hot-rolled samples and the samples after recrystallization annealing since it was not possible to measure the micro-hardness of thin cold-rolled samples. Each sample was measured at five points spaced 0.01 mm (10 measurements at each point) with a 25 gf load (values deviating from the mean by more than five units were not accounted for). Arithmetic means are used in the graphs and in the discussion.

To assess the effect of the alloying element content on the change in the size and number of large and fine particles, a study was carried out using a JEOL 6390A SEM scanning electron microscope (Akishima, Tokyo, Japan). Hot-rolled samples were used for the study. High-purity samples were preliminarily polished. The samples were examined at 300 (intermetallic compounds) and 10,000 magnifications (finely dispersed intermetallic compounds) after polishing. The content of chemical elements in the solid solution and large intermetallic compounds was determined using an EDAX energy-dispersive X-ray microanalyzer attachment.

Although the detected particles had different morphologies, for convenience, their average radius was determined. For this, the area of all objects in the study area was found. Then, the assumption was made that all objects had the shape of a circle. After that, the radius of each of them was found and then the average radius estimated. Taking into account the average radius, the total volume of these particles was found. The volume fraction of particles (*F_v_*) was calculated as the ratio of the volume of particles (equal to the number of particles on each side of the cube, multiplied by the average volume of these particles) to the volume in which they were measured.

The different types of recrystallization nuclei were estimated using the well-established method for aluminum alloys described in [9]. Data on the number of intermetallic particles were taken directly from the results of this study. Subgrain size data and their dependence on the Hollomon-Zener parameter were taken from [7] for 5182 alloys (4.8% Mg; 0.37% Mn; 0.15% Zn; 0.2% Ti; 0.060% Cu; 0.4% Si; 0.01% Fe), their chemical compositions being close to the investigated alloys. These data could be used because chemical composition change does not have a significant effect on magnesium-rich aluminum alloy subgrain size [39].

The retarding force of recrystallization *P_z_* was estimated using Formula (2) according to [40].

(2) $$P_{Z}=\frac{3\gamma_{B}F_{V}}{r_{D}}$$

where *F_V_* is the volume fraction of particles of the second phase, and *r_D_* is the average particle size.

The number of recrystallization nuclei depending on the Holomon-Zener parameter, including those formed by the PSN mechanism, was found according to the models proposed in [41,42].

## 3. Results and Discussion

### 3.1. Grain Structure

**Figure 1:**
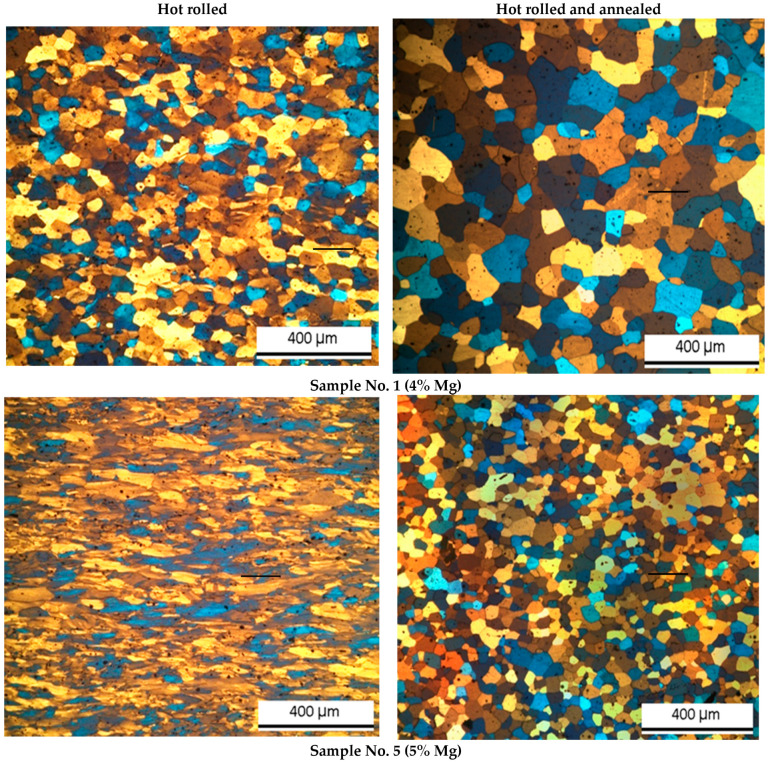

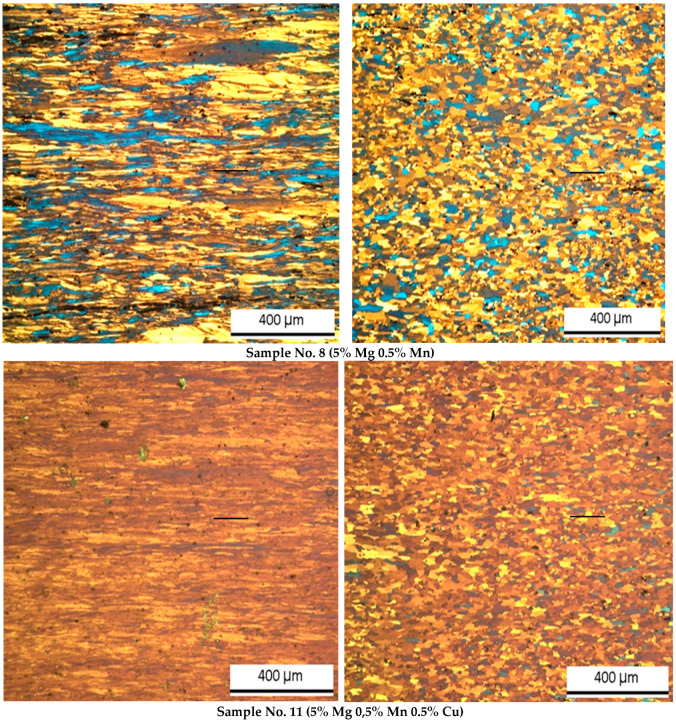
Grain structure of hot-rolled and annealed samples related to alloy chemical composition.

The grain size after recrystallization annealing decreased with increased magnesium content (Figure 1). This occurred because of two factors. The first was the development of an additional number of nuclei representing large Mg_2_Si-type intermetallic particles [28]. In addition, an increased number of nuclei was observed due to size decrease and, consequently, subgrain number increase, occurring inevitably with an aluminum alloying level increase [39]. Second, additional structure refinement occurred during crystallization. It should be also noted that a hot-rolled magnesium-rich alloy has a more elongated grain structure. In general, the grain size decreased with the addition of magnesium from 72 to 32 µm.

Manganese also contributes to grain refinement. During recrystallization, modification occurs due to increase of Al_6_(FeMn) nuclei number. Addition of up to 0.5% manganese increased the total amount of intermetallic particles by more than 20% and reduced the size of the grain structure to 25 µm.

In the case of copper addition, grain refinement during recrystallization is facilitated by development of CuMg_4_Al6-type particles [43]. Addition of 0.5% copper led to an increase of large intermetallic particles. Addition of 0.5% copper increased the total number of particles by seven times, and the grain size, in its turn, decreased to 15 µm. At the same time, a significant increase in the amount of finely dispersed intermetallic particles resulted in partial blocking of the recrystallization process.

It should be noted that in all the studied alloys after hot rolling, structural deformations were observed, as in [44,45,46,47]. However, in alloy No. 1, a recrystallized structure was observed. This can be explained by recrystallization, which occurs during cooling of a hot rolled workpiece. Recrystallization occurred in alloys No. 1 because it did not contain particles that inhibit the process. Grain growth during next annealing process can be explained by secondary recrystallization, which is also caused by the absence of fine particles [48].

**Figure 2:**
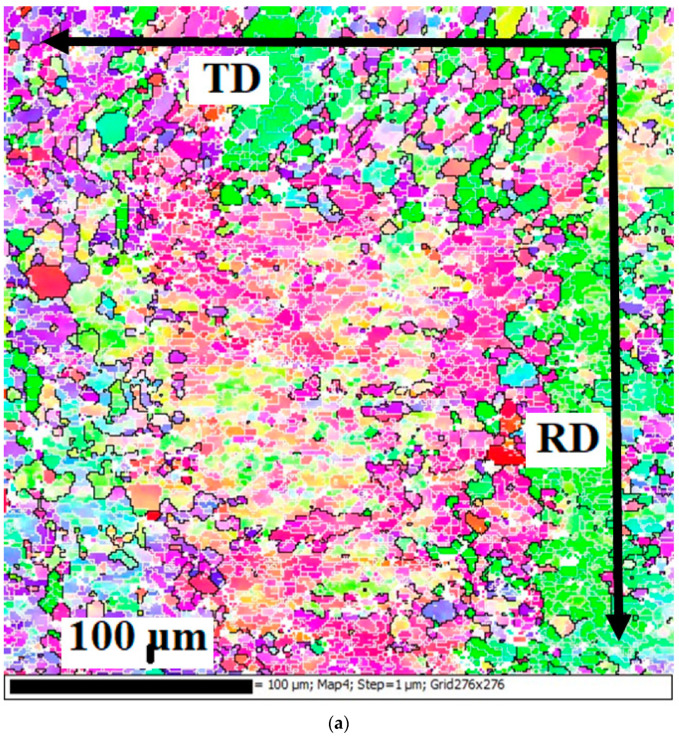

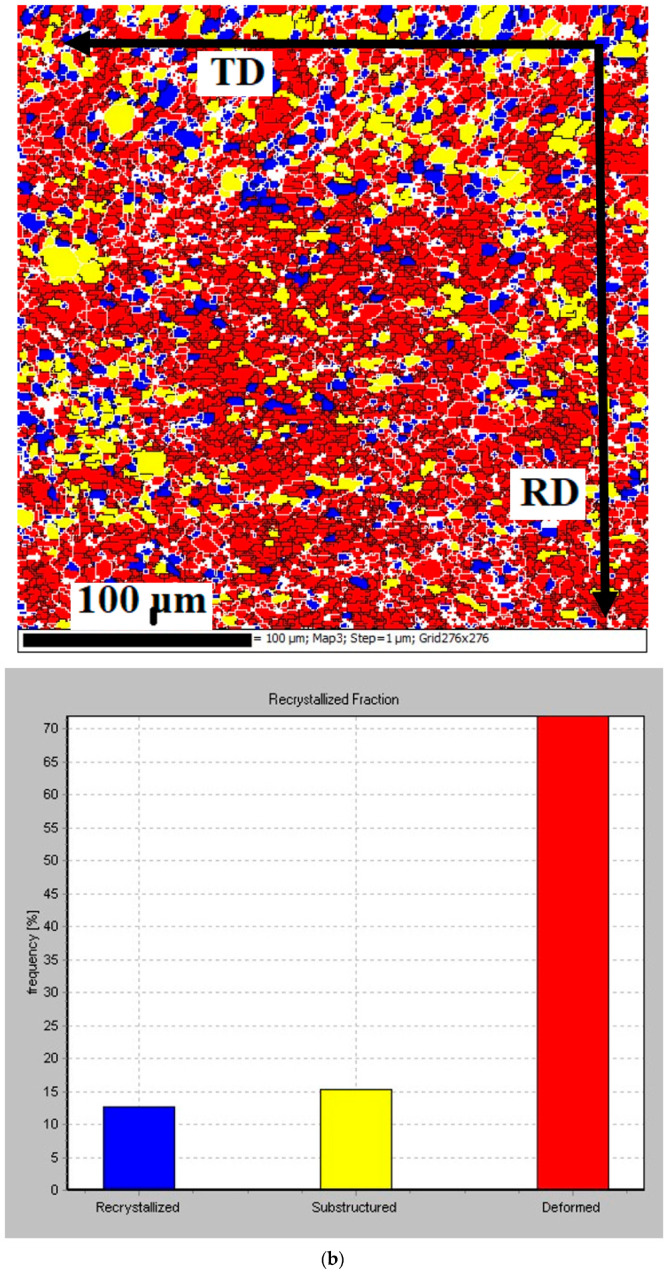

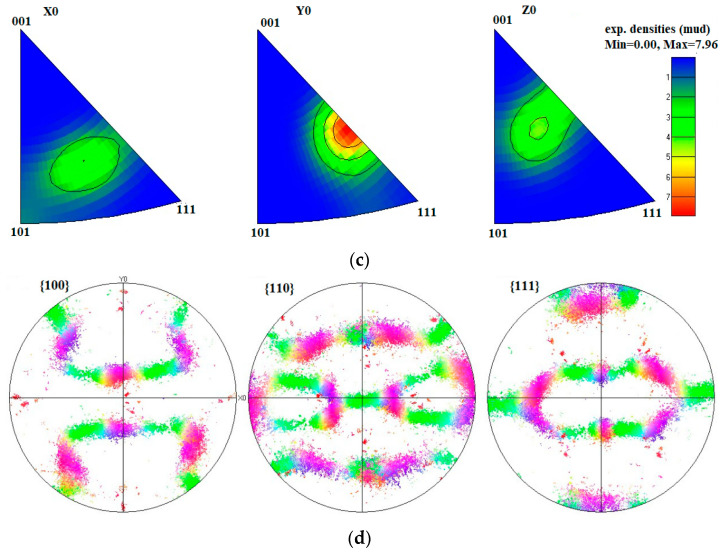
EBSD results for 5Mg0.5Mn0.5Cu alloy after hot-rolling: (**a**) subgrain structure; (**b**) fraction mapping; (**c**) inverse pole figure (**d**) pole figures.

**Figure 3:**
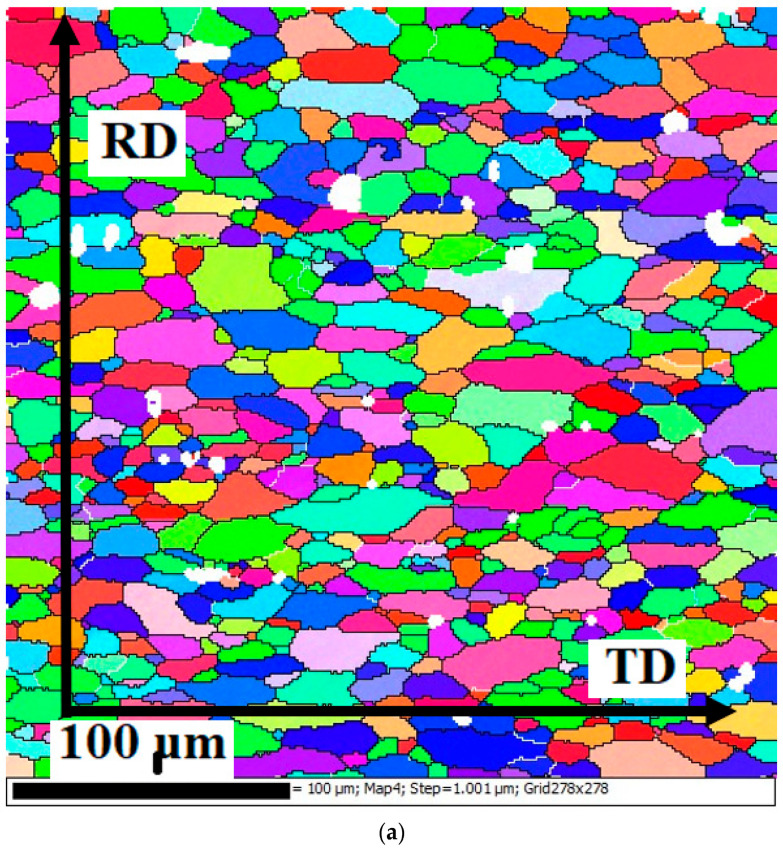

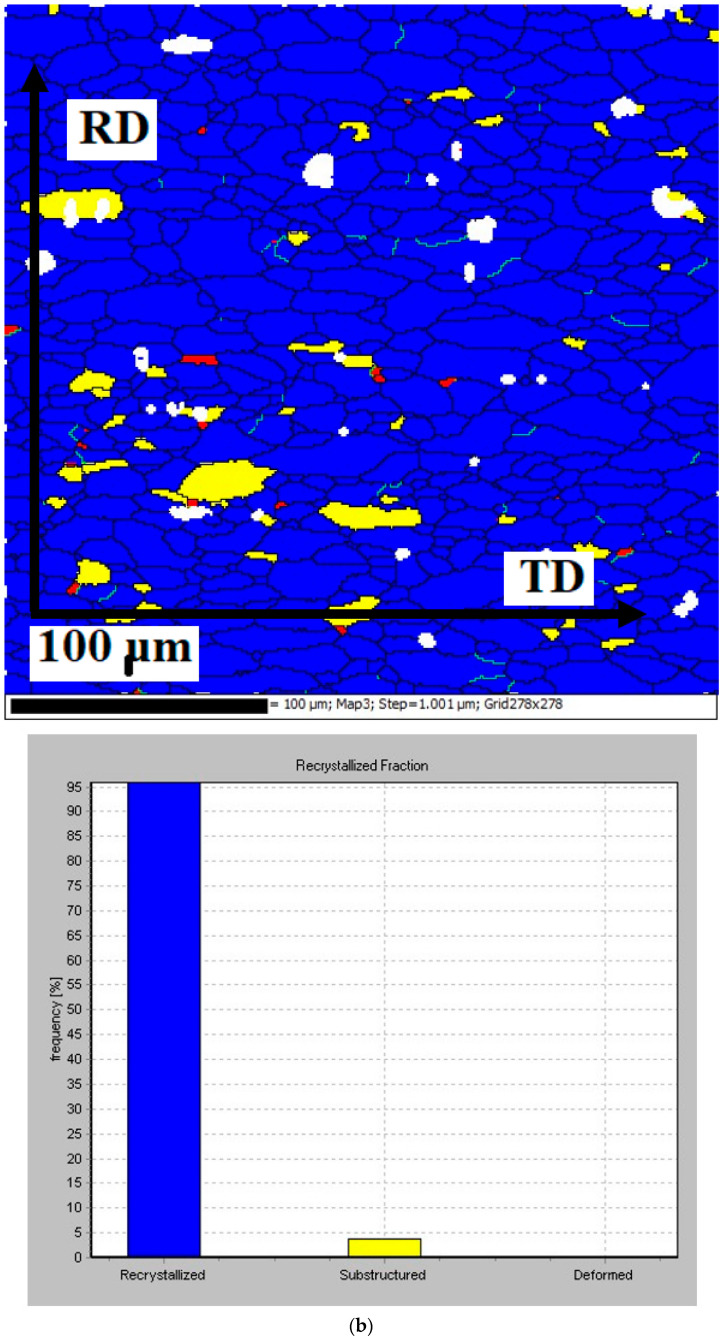

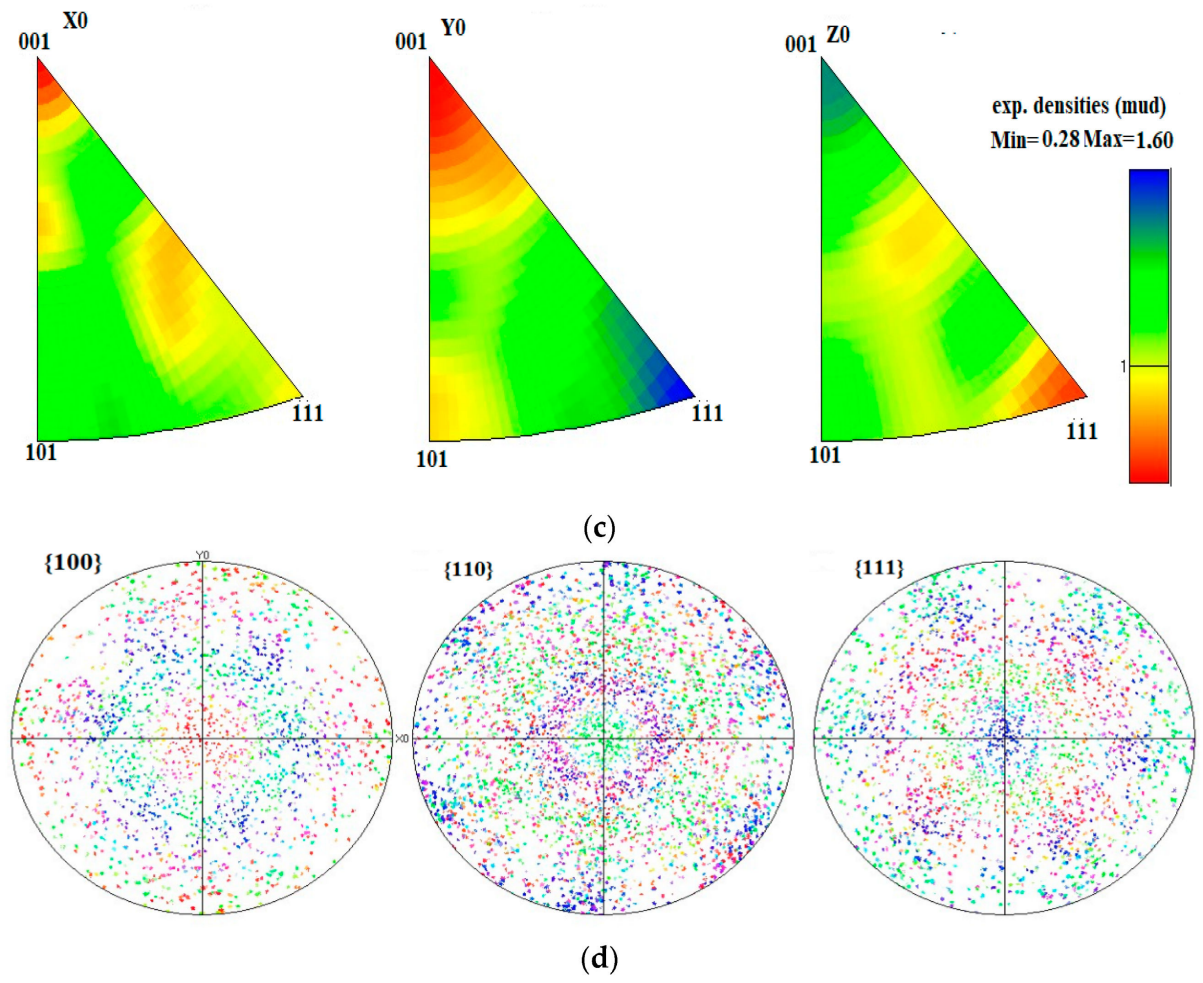
EBSD results for5Mg0.5Mn0.5Cu alloy after annealing: (**a**) subgrain structure; (**b**) fraction mapping; (**c**) inverse pole figure; (**d**) pole figure.

The results of EBSD in Figure 2 show that in the hot-rolled state, a structure consisting of deformed grains elongated in the direction of rolling was observed. It should be noted that, according to optical microscopy (Figure), the grain structure for this alloying was stretched in the rolling direction up to 500 µm, so the grain could not be fully represented. The grains consisted mainly of sections, and subgrains having a high dislocation density and a polygonized structure with an average size of 2 μm were formed within them, which is in good agreement with the data presented in [39]. The presence of small, well-formed subgrains, compared to low-alloyed aluminum alloys, can be explained by a decrease of stacking fault energies in high-magnesium alloys [49], which makes dislocations less mobile, and dislocations annihilate less quickly. In addition to areas with well-formed subgrains, there were areas with recrystallization nuclei. Their presence is explained by the fact that, despite the inhibition of recrystallization by finely dispersed particles and the fairly rapid cooling of the laboratory ingot in air, recrystallization nuclei had time for formation. Unfortunately, the method of obtaining an EBSD image did not make it possible to identify whether these nuclei were formed from subgrains or from particles. However, the presence of recrystallized areas indicated precisely their formation in the latter. The pole figure and the inverse pole (Figure 3c,d) showed classical rolling texture patterns [50]. At the same time, the set of crystallites for analysis in the hot-rolled state was very limited in the EBSD method. As result this could not provide the general pattern of texture distribution. Therefore, for a more detailed study of the texture, distribution-ray diffraction analysis is required, but was beyond the scope of this work.

The EBSD data for the 5Mg0.5Mn0.5Cu alloy in the annealed state in Figure 3 shows the presence of small-sized grains of 30 µm in which low-angle boundaries were not found. This, as well as the fact that the grains changed their shape and size, indicates that recrystallization was completed. Small inclusions of a non-recrystallized structure were observed. This suggests that recrystallization in these volumes was blocked by the action of finely dispersed intermetallic particles. In general, the EBSD data were in good agreement with the optical microscopy data. Based on analysis of inverse pole figures and pole figures, pronounced texture components were absent. This suggests that PNS is the dominant nucleation mechanism, since grains which grow during recrystallization from this nuclei type do not have of any texture components [28].

### 3.2. Micro-Hardness and Electrical Conductivity

**Figure 4:**
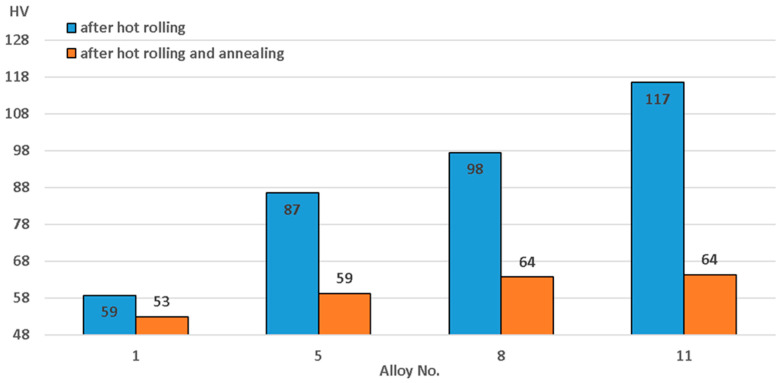
Changes in micro-hardness related to alloy chemical composition.

**Figure 5:**
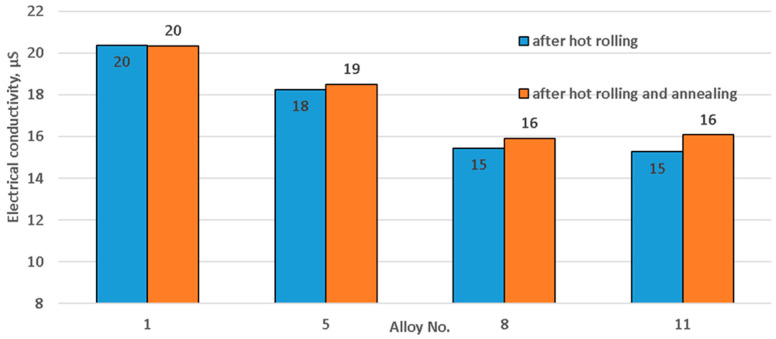
Change of electrical conductivity related to alloy chemical composition.

Measured micro-hardness and electrical conductivity results are presented in Figure 4 and Figure 5. A magnesium content increase from 4 to 5% resulted in micro-hardness increases from 58.72 to 86.52 HV and 52.92 from to 59.22 HV in the hot-rolled state and after annealing, respectively. Half a percent of manganese led to micro-hardness increases from 86.52 to 97.52 HV and from 59.22 to 63.72 HV in the hot-rolled state and after annealing, respectively. Copper led to micro-hardness growth from 97.52 to 114.42 HV, and after annealing and in the condition of hot-rolling it remained almost the same.

The greatest increase in hardness and, consequently, solid solution hardening, occurred when magnesium content increased. Manganese and copper did not have such a strong effect on this factor. The main mechanism of increase in micro-hardness with the addition of these elements was the presence of fine particles.

The micro-hardness difference in the hot-rolled and annealed states can be explained by the subgrain structure that was observed in the hot-rolled state. Subgrain structure in the annealed state would lead to a large number of dislocations, increasing micro-hardness [51]. Moreover, an increased amount of alloying elements, especially magnesium, always facilitates subgrain structure refinement [39]. Dislocation density, observed after annealing, is much lower [52] and reduces micro-hardness.

Electrical conductivity decreased in samples when the content of alloying components increased, regardless of the state (Figure 5). This occurred due to a large amount of alloying components melting in the supersaturated solid solution. In addition, during hot rolling, fine particles precipitate from the supersaturated solid solution. Therefore, subsequent annealing does not make much difference. An increase of magnesium content from 4 to 5% led to an electrical conductivity decrease from 20.4 to 18.3 μS. Adding 0.5% manganese reduced the electrical conductivity from 18.3 to 15.4 μS. Introduction of 0.5% copper led to an electrical conductivity decrease from 15.4 to14.8 μS.

### 3.3. Coarse Intermetallic Particles Study Using Scanning Microscopy

**Figure 6:**
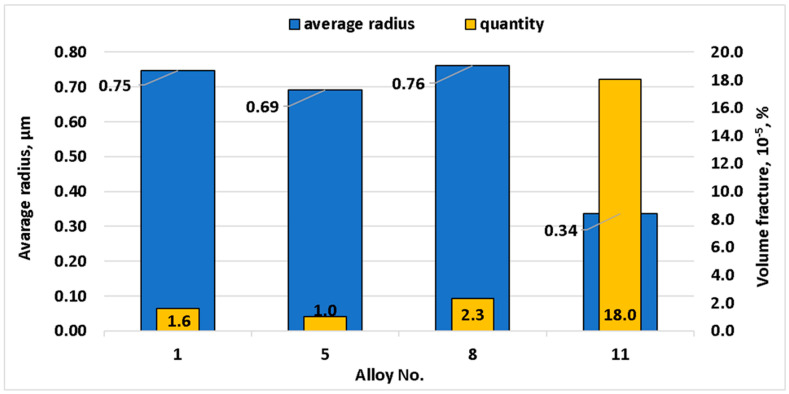
Changes in intermetallic compound size and number related to alloy chemical composition.

Coarse particle (intermetallic compounds) parameters measured using electron microscopy at the stage of hot rolling are shown in Figure 6. Magnesium and manganese did not have a large impact on intermetallic compound size and quantity. With the addition of 0.5% copper, the number of large intermetallic particles increased sevenfold, and their average radius decreased from 0.76 to 0.46 μm. The abovementioned changes occurred after copper was added and, as is shown below, this was caused by the development of a large amount of smaller Al_6_Cu_2_Mn_3_ intermetallic compounds. These particles formed due to copper poor solubility in aluminum matrix at the temperatures below 350 °C [53].

**Figure 7:**
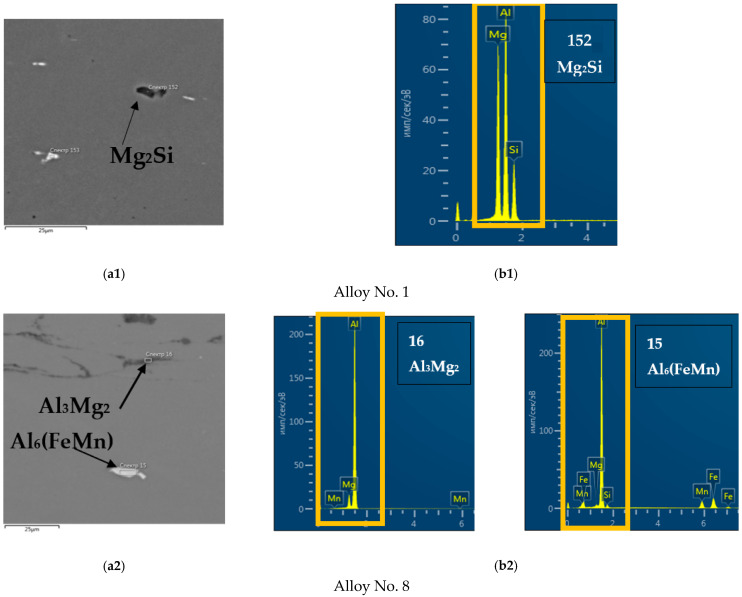

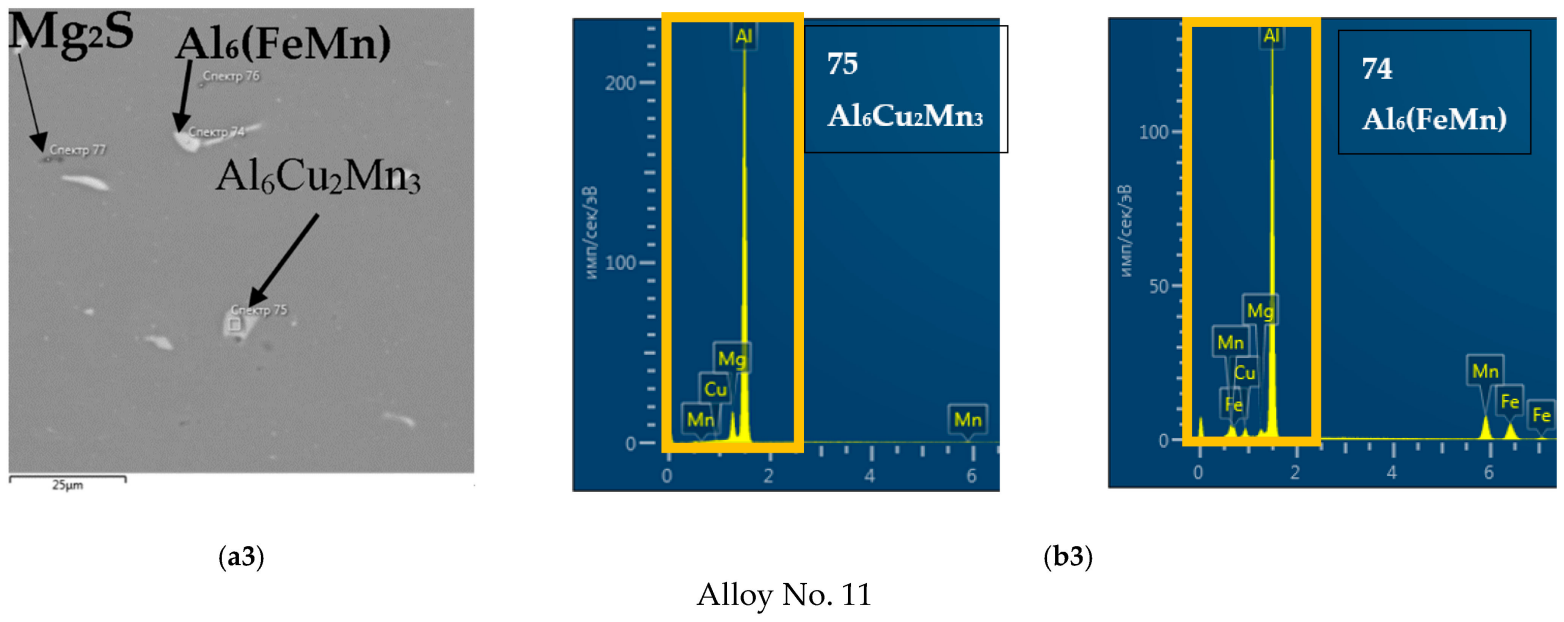
The types of intermetallic compounds (**a1**–**a3**); result of the EDS analyses (**b1**–**b3**).

The chemical compositions of intermetallic compounds are shown in Figure 7. The percentage of elements in each spectrum are shown in Table 3. Elements such as Fe and Si were identified in all samples. These elements were not added to the alloy and were present as impurities contained in the aluminum ligature.

**Figure 8:**
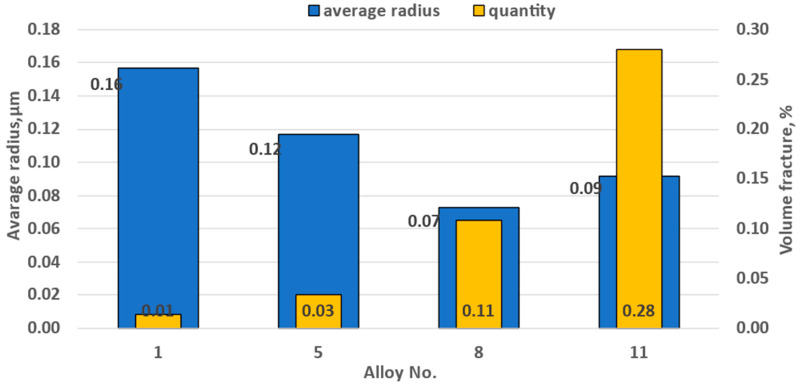
Change of dispersoid size and number depending on alloy chemical composition.

The main particles in magnesium alloys consisted of Mg_2_Si. They had an elongated shape and average size of about 5–10 µm (Figure 8). Based on the presence of magnesium and silicon in them, it can be assumed with a high degree of probability that these particles were, indeed, Mg_2_Si [53,54], and they often appear in high-magnesium alloys [33,55]. These particles are primary intermetallic compounds that “survived” the process of homogenization. EDX analysis showed the presence of aluminum in these alloys, which, however, was a consequence of the influence of the solid solution involved in this research method, as, for example, in [33,54,56]. The supersaturated solid solution contained magnesium, which explains strengthening of the solid solution (Figure 4) and the decrease of electrical conductivity (Figure 5).

When manganese was added, particles appeared which, in terms of the content of chemical elements, appeared to be Al_6_FeMn, which is common in these types of alloys [34,57,58]. They had a 10–15 microns size and elongated shape. Al_8_Mg_5_ type particles, typical for magnesium-rich aluminum alloys, were found in [33,35,59]. The absence of such phases in alloys 1–5 can be explained by the limited area where large intermetallic particles chemical analysis was performed. In addition, Mg_2_Si and Al_3_Si particles are similar in color, making it difficult to select zones where both types of particles are present.

Al_6_(FeMn) type particles appeared in alloys 6–8 due to manganese addition. As can be seen in Figure 7, the solid solution also contained manganese.

Intermetallic compounds can be identified by chemical composition as phases such as Mg_2_Si and Al_6_(FeMn). There was a phase close to Al_20_Cu_2_Mn_3_ observed in alloys with joint manganese-copper alloying [60,61]. Average sizes of intermetallic compounds were 3–5, 5–8 and 10–15 µm, respectively. Mg_2_Si particles were represented by elongated dark inclusions, Al_6_(FeMn) particles were light and had elongated shape, and Al_20_Cu_2_Mn_3_ particles were light square-shaped particles. Manganese, copper and magnesium were also observed in a supersaturated solid solution.

Note that Fe and Si impurities, despite their rather low content, play an important role by participating in the formation of such particles as Mg_2_Si and Al_6_(FeMn). Therefore, our data are relevant for alloys with the usual level of impurity elements. Additional research on the relationship between intermetallic particles and the recrystallization process in alloys made from high purity master alloys may be needed to account for the effect of Si and Fe reduction.

### 3.4. Fine Particle Studies using Scanning and Transmission Microscopy

Result of fine particle electron microscopy investigations are shown in Figure 8. After magnesium content reached 5%, the size of the dispersoids decreased from 0.16 to 0.12 μm, and their number increased three times. Addition of 0.5% manganese led to reduction of their radius from 0.12 to 0.07 μm, and their quantity increased more than 3.66 times. Addition of 0.5% copper increased manganese dispersoid radius from 0.07 to 0.1 μm, the number grew more than three times. As a result, copper had most prominent impact on recrystallization during hot rolling.

**Figure 9:**
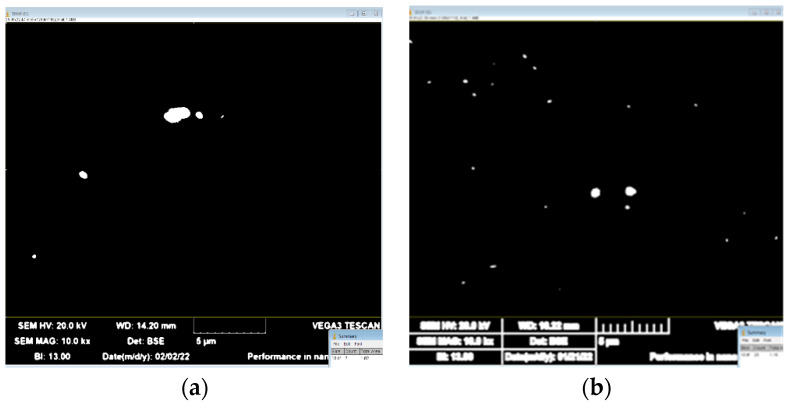
Scanning electronic microscopy of (**a**) 5Mg and (**b**) 5Mg0.5Mn alloys.

**Figure 10:**
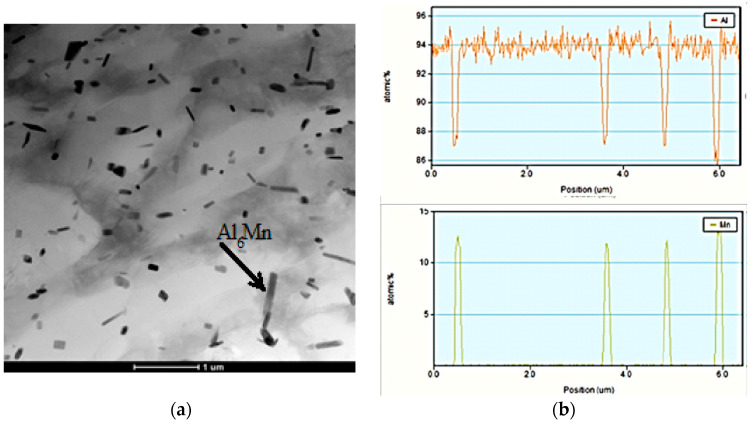

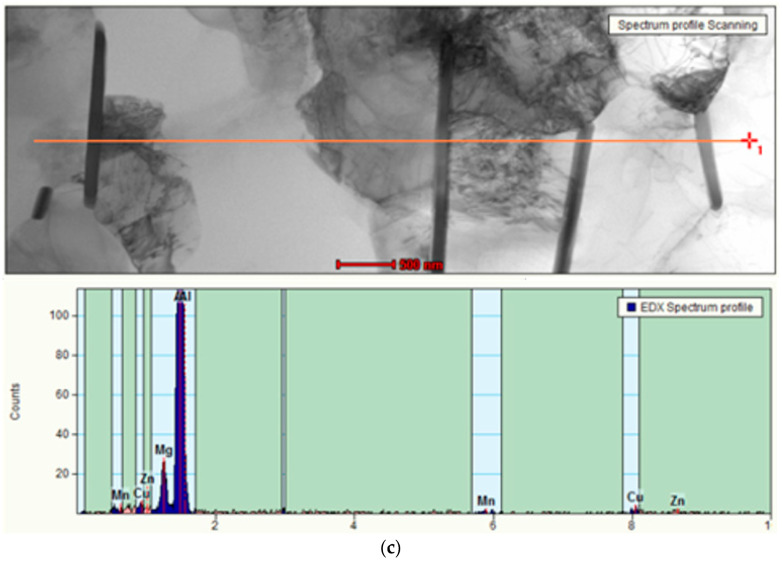
Transmission electronic microscopy of the 5Mg0.5Mn alloy (**a**); EDS results for the 5Mg0.5Mn alloy (**b**,**c**).

It should be noted that very few dispersoids are present in alloys containing magnesium only. They were detected by SEM (Figure 9), where it was possible to study larger areas, but this method does not allow their chemical composition to be analyzed. However, it should be noted that such a small number of particles has practically no effect on the recrystallization process, so there is no need for the laborious process of detection using transmission microscopy. As the proportion of manganese increased, the number of finely dispersed particles increased also (Figure 9). At the same time, manganese was the main element found in these particles. In addition, there were particles containing both manganese and iron, which from the chemical composition consisted of Al_6_Mn and Al_6_FeMn [62,63] (Figure 10). The presence of magnesium in the EDS results can be explained by solid solution impact. Although the particle number increased, and the size decreased, these particles did not have a decisive influence on the course of the recrystallization process.

**Figure 11:**
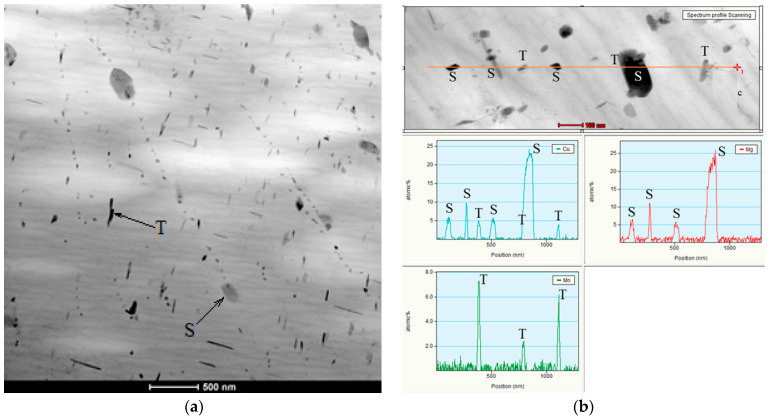
Transmission electronic microscopy of the 5Mg0.5Mn0.5Cu alloy (**a**). EDS results for the 5Mg0.5Mn0.5Cu alloy (**b**).

**Figure 12:**
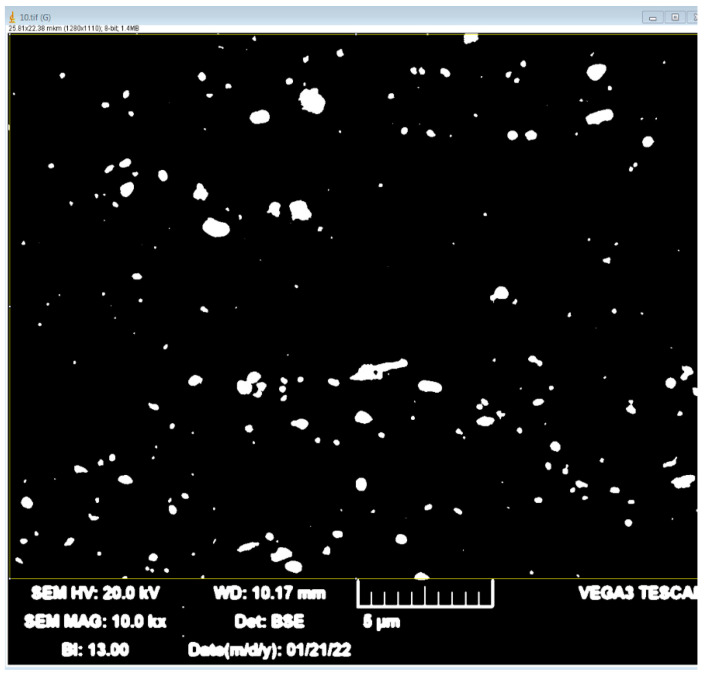
Scanning electronic microscopy of the 5Mg0.5Mn0.5Cu alloy.

TEM analysis made it possible to reveal particles in the form of plates close in composition to the S Al_2_CuMg phase, since the Cu:Mg ratio in them was close to 1:1 [62,63], and the T of which did not exceed 300 nm. In addition, there were plates that could be described as the T(Al_20_Cu_2_Mn_3_) phase, since the Mn:Cu ratio was close to 3:2 [64,65] (Figure 11). The size of this phase was not more than 100 nm. It should be noted that these phases are the main blockers of the recrystallization process. At the same time, as copper content grew, the number of fine particles increased, and the retarding force of recrystallization also increased. It should be noted that although the S phase in the form of S” can be strengthening, in this case, as in the T phase, strengthening occurred only due to the inhibition of recrystallization. It is necessary to carry out hardening and subsequent artificial aging at lower temperatures to obtain S”. The particles close to S and T observed in this case were most likely formed during the decomposition of a supersaturated solid solution, which occurred during hot rolling and annealing at temperatures of 360–500 °C; therefore, by their nature, they should be close to equilibrium. In this way, with copper addition, new particles formed, so that the total number significantly increased as confirmed by the SEM result (Figure 12).

### 3.5. Calculations of the Effect of the Size and Number of Intermetallic Particles on the Inhibition Force and Nucleation during Recrystallization

**Figure 13:**
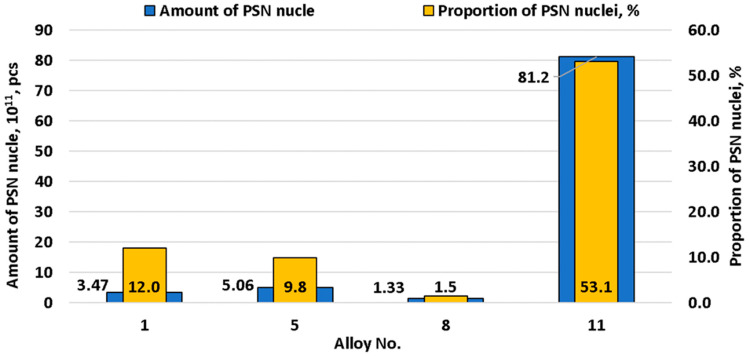
Change of PSN nuclei number depending on alloy chemical composition.

Figure 13 shows the effect of chemical composition on the number of nuclei for recrystallization which proceeds based on the particle stimulated nucleation (PSN) mechanism. When the amount of alloying elements was increased, a slight increase in PSN nuclei number (until copper was added to the alloy) was observed. At the same time, the proportion of PSN nuclei of the total nuclei number did not change.

**Figure 14:**
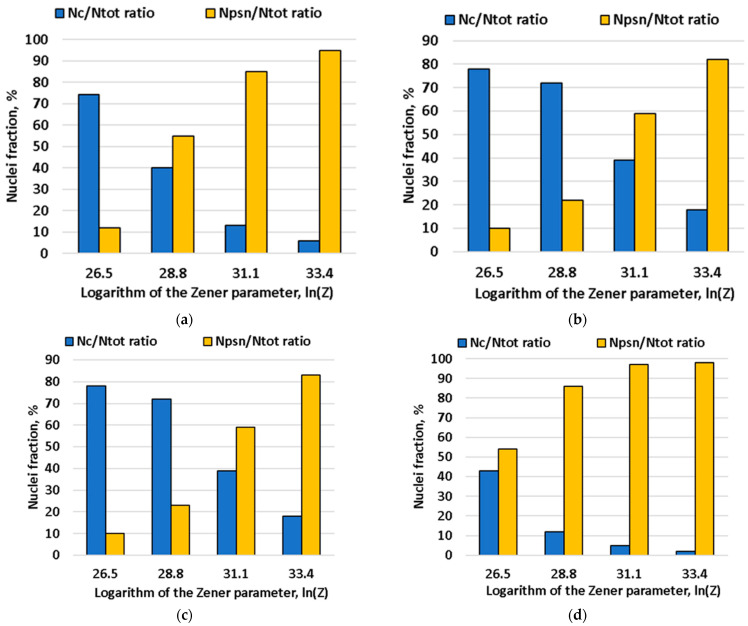
Changing t of N_c_ subgrains-based nuclei and N_PSN_ second-phase particles-based nuclei fraction depending on Zener-Hollomon parameter and alloy chemical composition: (**a**) alloy No. 1, (**b**) alloy No. 5, (**c**) alloy No. 8, (**d**) alloy No. 11.

Figure 14 shows the effect of chemical composition and the Zener-Hollomon parameter on the number of nuclei formed from PSN or from subgrains. Subgrains are the predominant source of nucleation at low Zener-Hollomon parameter values. However, as Zener-Hollomon parameter increased, the subgrain size rapidly decreased and particle-based nucleation becomes predominant. Particle-stimulated nucleation prevailing over subgrain-based nucleation is generally typical for magnesium-rich aluminum alloys. This is caused, primarily, not by the large size and number of second-phase particles, but by small-sized subgrains in this type of alloy [30,66]. This results in intense crystallographic hardening and lower stacking fault energy compared to other alloys [67]. The ratio of subgrain-based and second-phase particles-based nuclei numbers remained almost unchanged before the addition of copper to the alloy. This was associated with an insignificant increase of intermetallic compound size and number. However, their number spiked after copper was added. As a result of this chemical composition, second-phase particles became the main nuclei sources during recrystallization. Thus, in terms of recrystallization, the studied alloy nuclei development mechanism was close to that of the 1565ch alloy, where intermetallic particle-based nucleation prevails over subgrain-based nucleation [28]. It must be mentioned that magnesium and manganese did not have a pronounced impact on PSN nuclei, but the number of nuclei from intermetallic compounds grew 4.4 times after copper was added.

**Figure 15:**
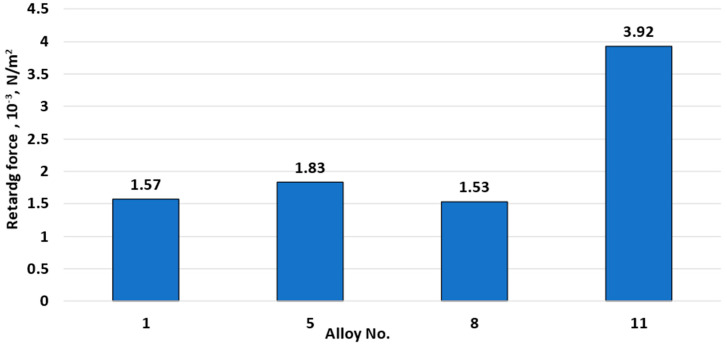
Changes in the recrystallization retarding force depending on alloy chemical composition.

Figure 15 shows the recrystallization retarding force. The retarding force of recrystallization did not change significantly when the content of magnesium and manganese increased. This was because the number of fine particles did not grow significantly with increasing magnesium and manganese content (Figure 7). However, with 0.5% copper addition, dispersoids increased dramatically, leading to an increase of the recrystallization retarding force from 1.53 × 10^−3^ N/m^2^ to 3.92 N/m^2^ (Figure 8). Zirconium produced an even greater significant effect on the recrystallization retarding force. The results, presented in Figure 8 correlate with the optical microscopy data (Figure 1), demonstrating partial blocking of the recrystallization process due to copper addition.

## 4. Conclusions

Increasing magnesium, manganese and copper content affected grain size due to an increase in nuclei number as a result of large intermetallic particle development. The main large intermetallic particles were Mg_2_Si, Al_6_(FeMn) and Al_20_Cu_2_Mn_3_.

In the alloys containing no other alloying components, apart from magnesium, the level of finely dispersed components was very low. When manganese was added, Al_6_Mn and Al_6_(FeMn) types dispersoids appeared, and when copper was added, fine particles close to T(Al_20_Cu_2_Mn_3_) and S(Al_2_CuMg) appeared. Copper addition significantly increased the total number of intermetallic particles and dispersoids, leading to recrystallization retarding force intensification. Therefore, copper addition partially blocked this process.

Nucleation modeling during recrystallization showed that this alloy (like other magnesium alloys) is prone to PSN mechanism activation. However, subgrain-based recrystallization nuclei development prevailed at low values of the Zener-Hollomon parameter without copper addition. The PSN mechanism began to prevail when copper was added, even at low Zener-Hollomon parameter values.

## Figures and Tables

**Table 1:** Chemical compositions of the investigated Al-Mg alloys.

Content of Elements, %					
Alloy No.	Mg	Mn	Cu	Si	Fe
1	4	-	-	<0.05	<0.05
2	4.25	-	-	<0.05	<0.05
3	4.5	-	-	<0.05	<0.05
4	4.75	-	-	<0.05	<0.05
5	5	-	-	<0.05	<0.05
6	5	0.2	-	<0.05	<0.05
7	5	0.35	-	<0.05	<0.05
8	5	0.5	-	<0.05	<0.05
9	5	0.5	0.1	<0.05	<0.05
10	5	0.5	0.25	<0.05	<0.05
11	5	0.5	0.5	<0.05	<0.05

**Table 2:** Ingot hot rolling schedule.

Pass No.	Initial Thickness, mm	Final Thickness, mm	Thickness Reduction, %
1	30	27	10
2	27	24	11.1
3	24	21	12.5
4	21	19	9.5
5	19	17	10.5
6	17	15	11.8
7	15	13	13.3
8	13	12	7.7
9	12	11	8.3
10	11	10	9.1
11	10	8	20
12	8	6	25
13	6	4	33.3
14	4	3	25

**Table 3:** Content of elements in particles.

Spectrum	Particle	Elements Content, %					
		Al	Mg	Mn	Cu	Fe	Si
15	Al_6_(FeMn)	69.23	0.64	10.6	-	17.55	1.98
16	Al_3_Mg_2_	67.50	32.29	-	-	-	0.22
74	Al_6_(FeMn)	68.09	1.08	15.73	3.93	11.17	-
75	Al_6_Cu_2_Mn_3_	75.10	0.65	12.01	0.52	11.72	-
152	Mg_2_Si	51.73	25.94	-	-	-	22.33

## Data Availability

The data presented in this study are available on request from the corresponding author.
